# Shared Decision Making in Patients with Stable Coronary Artery Disease: PCI Choice

**DOI:** 10.1371/journal.pone.0049827

**Published:** 2012-11-30

**Authors:** Megan Coylewright, Kathy Shepel, Annie LeBlanc, Laurie Pencille, Erik Hess, Nilay Shah, Victor M. Montori, Henry H. Ting

**Affiliations:** 1 Division of Cardiovascular Diseases, Department of Medicine, Mayo Clinic, Rochester, Minnesota, United States of America; 2 Knowledge and Evaluation Research Unit, Mayo Clinic, Rochester, Minnesota, United States of America; 3 The Section of Creative Media at Mayo Clinic, Rochester, Minnesota, United States of America; 4 Division of Emergency Medicine Research, Department of Emergency Medicine, Mayo Clinic, Rochester, Minnesota, United States of America; 5 Division of Health Care Policy and Research, Department of Health Sciences Research, Mayo Clinic, Rochester, Minnesota, United States of America; 6 Division of Endocrinology, Diabetes, Metabolism, and Nutrition, Department of Medicine, Mayo Clinic, Rochester, Minnesota, United States of America; Sapienza University of Rome, Italy

## Abstract

**Background:**

Percutaneous coronary intervention (PCI) and optimal medical therapy (OMT) are comparable, alternative therapies for many patients with stable angina; however, patients may have misconceptions regarding the impact of PCI on risk of death and myocardial infarction (MI) in stable coronary artery disease (CAD).

**Methods and Results:**

We designed and developed a patient-centered decision aid (PCI Choice) to promote shared decision making for patients with stable CAD. The estimated benefits and risks of PCI+OMT as compared to OMT were displayed in a decision aid using pictographs with natural frequencies and text. We engaged patients, clinicians, health service researchers, and designers with over 20 successive iterations of the decision aid, which were field tested during real-world clinical encounters involving clinicians and patients. The decision aid is intended to facilitate knowledge transfer, deliberation based on patient values and preferences, and shared decision making.

**Conclusions:**

We describe the methods and outcomes of the design and development of a decision aid (PCI Choice) to promote shared decision making between clinicians and patients regarding the choice of PCI+OMT vs. OMT for treatment of stable CAD. We will evaluate the impact of PCI Choice on patient knowledge, decisional conflict, participation in decision-making, and treatment choice in an upcoming randomized trial.

## Introduction

Percutaneous coronary intervention (PCI) does not lower the risk of death or myocardial infarction (MI) for patients with stable coronary artery disease (CAD) when added to optimal medical therapy (OMT) [1], although PCI is associated with more rapid improvement in symptoms [2], [3].

Misconceptions exist among patients regarding the potential benefit of PCI+OMT for stable CAD as nearly 90% of patients in a recent study believed that PCI reduces the risk of MI [4]. The selection of PCI+OMT vs. OMT alone for stable CAD represents a preference-sensitive decision where comparable, alternative treatments exist. Shared decision making may improve patient knowledge and involvement in decision-making to promote an “informed, values-based choice among two or more medically reasonable alternatives.” [5]

Clinicians want to “do the right thing” for patients with stable CAD, using professional society guidelines and appropriate use criteria to assist in decision making [6]. Often missing, however, are the skills and tools to best involve patients in a decision making that reflects patient goals and preferences. In this paper, we describe the process of designing and testing a decision aid for the treatment of stable CAD to address these gaps for patients in whom a clinical choice exists between OMT or PCI+OMT. The decision aid is intended for use following stress testing and upstream from diagnostic angiography; if diagnostic angiography is performed, the minority of patients in whom a choice of surgery is then relevant would no longer utilize the decision aid, as this choice is not modeled. The process of decision aid creation included review of pertinent literature; design and development of the decision aid with input from patients, clinicians, designers and researchers; and the testing of successive iterations during real-world clinical encounters.

## Methods

**Figure 1:**
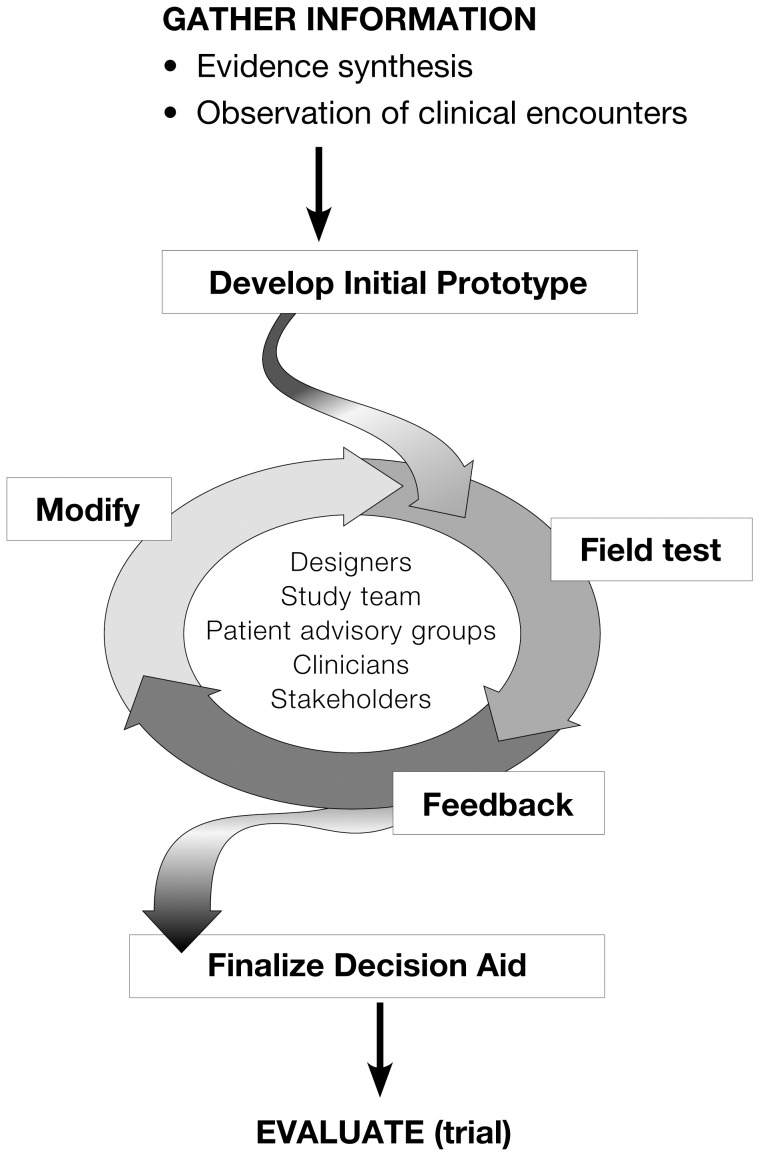
Process for development and prototyping of decision aid.

We used a practice-based, patient-centered, and participatory approach to design PCI Choice [7], [8], [9], [10], [11], requiring multidisciplinary input from clinicians (cardiologists, cardiology fellows, nurse practitioners, physician assistants, and nurses), health service researchers, design experts, statisticians, and patients. The process of decision aid design and development involves 1) review and synthesis of the available evidence and its endorsement by stakeholders; 2) analysis of usual practice; 3) development of an initial prototype; 4) field testing of the prototype in clinical settings under the study team's supervision; and 5) successive iterations and further testing of the prototype (Figure 1). The resulting decision aid is intended to be nondirective, encouraging clinicians to create a conversation with patients using their own communication styles, while simultaneously ensuring that key information is conveyed and that patient preferences are elicited [12]. We required sufficient evidence of ease of use and clarity of information from our study team, participating clinicians, and patients prior to selecting a final prototype. We tested the decision aid within the cardiovascular division at Mayo Clinic Rochester, which is comprised of 150 staff cardiologists (including 15 interventional cardiologists), 40 cardiovascular fellows, and approximately 700 allied health staff, including nurse practitioners, physician assistants, and specialized cardiac catheterization nurses. The outpatient cardiology practice is divided into 18 subspecialty clinics; prototypes of PCI Choice were tested in two clinics that see the highest volume of patients with stable CAD.

### Methods Step 1: Review and synthesis of the evidence

Synthesis of the evidence for the treatment of stable CAD was conducted by an interventional cardiologist (H.H.T.) and cardiology fellow (M.C.), up to date as of April 2012; we performed a detailed PubMed search, referenced American College of Cardiology/American Heart Association guidelines, and reviewed relevant bibliographies. The content of the decision aid was then vetted with cardiologists and cardiology fellows through a grand rounds presentation, focus groups, and individual interviews. Experts in the field of outcomes research in stable CAD at outside institutions were also included in reviewing the selected evidence base.

The study was approved by the Institutional Review Board (IRB) at Mayo Clinic-Rochester. All participants provided their verbal informed consent, as prespecified in our protocol submitted to, and approved by, the IRB. Documentation of the verbal script used to obtain consent was also submitted and approved by the IRB. Verbal consent was utilized given the minimal risk nature of the study in which a decision aid was being field tested and revised with feedback from patients involved. Verbal consent was obtained by trained study personnel involved in the testing of the decision aid, and receipt of verbal consent was documented within a spreadsheet that contained the name and medical record number of all patients considered for eligibility. This spreadsheet was distinct from the de-indentified database that included patient observations and feedback with use of the decision aid. Patients were not asked to submit written documents or complete surveys as part of this protocol.

### Methods Step 2: Analysis of usual practice

Members of the study team undertook an in-depth evaluation of the usual flow of patient care at Mayo Clinic Rochester for patients with stable CAD. Outpatient clinical visits with both cardiovascular fellows and staff cardiologists were observed by study personnel to identify and document usual care patterns. Further observations were performed of specialized cardiac catheterization lab nurse interactions with patients in the outpatient setting in preparation for catheterization, and during the day of the procedure in the prep area of the catheterization lab. Lastly, observations were made in catheterization lab during the procedure and in the recovery room after the procedure. Multiple interactions in each area of care were observed to create a description of routine usual care patterns, in addition to direct observation of several patients from start to finish (starting with the initial cardiology consultation to the diagnostic catheterization and/or PCI procedure). Formal input was gathered from stakeholders regarding timing of the decision aid in relation to coronary angiography during a cardiovascular grand rounds presentation, as well as focus group sessions including cardiovascular fellows and staff, catheterization lab nurses, clinical assistants, nurse practitioners and physician assistants, and patients and their families.

### Methods Step 3: Development of an initial prototype

Content experts (H.H.T, M.C.) and designers (K.S.) partnered to create the first iteration. Careful consideration of how to display numerical estimates of risk and benefit is integral to the process. The preferred method in risk communication is the use of pictographs, which specifically includes display of the proportion of patients who do not receive any benefit from the proposed treatment, as well as those that do benefit [13]. For example, we include language such as, “Out of 100 people, 60 will experience benefit, and 40 will not.” The use of natural frequencies with a common denominator may be clearer to patients than communicating in relative risks [14]. We have found that using pictographs to display absolute risk improves communication of personalized benefit [15], and is effective across diverse sociodemographic groups [16].

### Methods Step 4: Field testing

Field testing began with patient advisory groups with experience in decision aid development, which included the long-standing Diabetes Research Advisory Group (DAG), comprised of 15–20 community members with diabetes who meet with Mayo Clinic researchers on a monthly basis to provide feedback on research proposals and activities, as well as the Cardiovascular Patient and Family Advisory Council (comprised of over 25 patients and family members). The groups evaluated the decision aid early in the process with one-time focused meetings and reconvened to review our final prototype.

### Methods Step 5: Successive iterations

A critical method in decision aid development is testing of successive iterations, with content and format adjustment based on clinical observations in real world clinical encounters. Study team members observed clinicians delivering the prototypes to patients with stable CAD. Clinical interactions were evaluated for ease of use and fit within flow of care; patient and clinician body language; and content of discussion. Our interest was in shifting the current technical dialogue (e.g., “Based on my experience, I recommend that you undergo PCI to treat a 70% blockage”) to a conversation between the clinician and patient regarding the patient's health care goals (“Let's discuss what is important to you and alternative choices for treatment”). We have found that this shift in approach led to increased patient knowledge, decreased decisional conflict, and increased medication adherence [17]. After each clinical observation, the decision aid was revised by our development team over the course of 1–2 weeks. The process was repeated until there were consistent observations of knowledge transfer and elicitation of patient preferences.

## Results

### Results Step 1: Review and synthesis of the evidence

The 2011 revised ACC/AHA guidelines recommend PCI for stable CAD when symptoms persist while on OMT; [18] this is also reflected in the 2009 Appropriateness Criteria for Coronary Revascularization [19]. These recommendations are in part based upon COURAGE, a large, randomized trial demonstrating no difference in rates of MI or death for PCI+OMT over OMT in stable angina [20]. COURAGE was prefaced by years of data comparing PCI+OMT vs. OMT for stable CAD with similar results [21].

Quality of life data in the COURAGE trial [3], and other randomized trials of revascularization for stable angina such as BARI 2D [2], demonstrated an initial benefit of PCI+OMT early on for symptom relief which waned over time. We examined symptom-stratified quality of life data from COURAGE, creating two unique decision aids: one modeled Canadian Cardiovascular Class (CCS) Class I/II (mild) angina, and the second, CCS Class III (moderate) angina. The benefit of PCI+OMT is more dramatic in those patients with greater symptoms: for example, of patients with moderate angina (Class III) who chose PCI+OMT, 76% saw clinically significant improvement in their quality of life at one month as compared to 57% with mild symptoms (Class I/II) [3].

Large registries and clinical trials examining outcomes such as bleeding, death, and stent thrombosis provided data for risk estimates. A recent analysis of several trials of elective PCI demonstrated a 2% risk of periprocedural bleeding [22]. The risk of longer term bleeding (one year) was based on a registry of over 80,000 patients in which the risk of a major bleed with aspirin alone was 4% per patient-year, with the addition of clopidogrel raising the risk to 7% per patient-year [23]. We utilized data from COURAGE on need for revascularization with an initial strategy of OMT (14%) [20] and more recent data on restenosis risk in the drug-eluting stent era (7%) [24].

In PCI Choice, we specifically modeled population estimates for risk as opposed to personalized predictions for two reasons: 1) less clinician burden by eliminating the need to enter clinical variables or print a unique copy of the decision aid and 2) consistent observations that once patients understood there was no mortality benefit to PCI+OMT, risk estimates had little impact on decision making; instead, we observed that the severity of symptoms drove patient choice.

### Results Step 2: Analysis of usual practice

**Figure 2:**
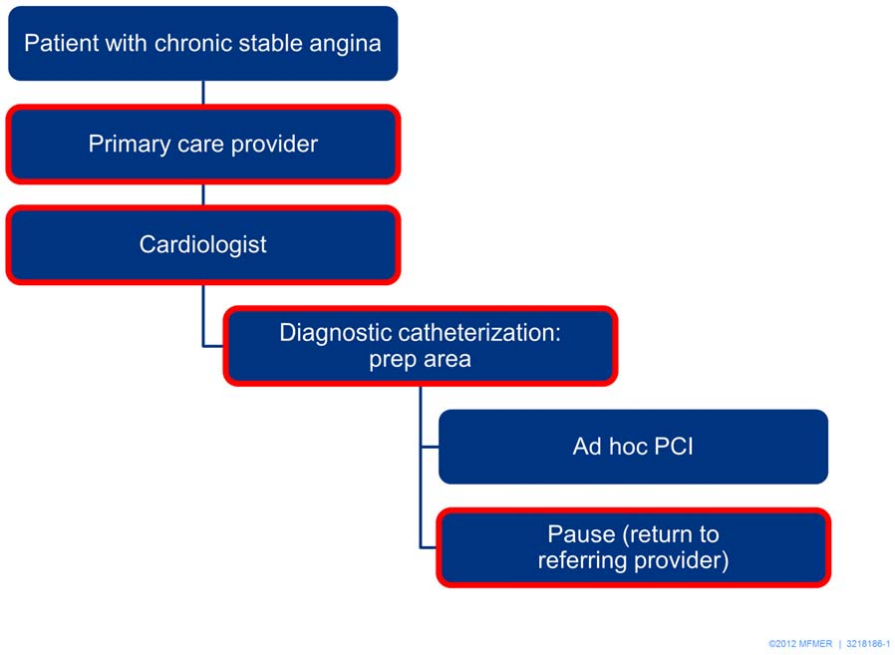
Flow of care for patients with chronic stable angina; red border indicates potential for shared decision making.

Two clinicians (H.H.T. and M.C.) mapped the flow of care for patients with stable CAD at Mayo Clinic-Rochester, identifying potential points in time conducive to shared decision making regarding the choice of PCI vs. OMT. (Figure 2) Patients with chronic stable angina are typically first seen by a primary care provider, and are then referred to a cardiologist for consideration of PCI with or without a preceding stress test. Stress tests are often performed prior to PCI at some point in the flow of care. The majority of PCIs are performed ad hoc – that is, PCI is performed immediately following a diagnostic coronary angiogram on a sedated patient without a pause to discuss the benefits and risks of alternative treatment options. While the majority of PCI performed in our institution is ad hoc in the setting of unstable angina or myocardial infarction, PCI for stable CAD is also performed ad hoc (266/322; 83%). This trend is seen nationally; for example, in a recent publication from the comprehensive New York State database, when excluding patients with MI in the preceding 24 hours, more than 80% of PCI procedures were performed ad hoc [25].

After discussions with cardiologists, nurses, patients, and our development team, the consensus was that the optimal time for shared decision making in our practice is upstream from diagnostic catheterization during the clinical encounter between the cardiologist and the patient, accommodating the current practice of ad hoc PCI. We also recognize that some of the shared decision making may begin during the visit with the primary care provider, and that opportunities exist in the prep area of the catheterization lab as well as during a potential pause following diagnostic catheterization for shared decision making.

In a typical clinical encounter within the cardiology specialty clinics, prior to referral for coronary angiography, patients describe their symptoms and experience with medications to the clinician, undergo a physical examination, and then review pertinent testing, including stress testing, with their clinician. A computer may be used to display results of testing, with the clinician sitting at a desk and the patient sitting on an adjacent couch. If ischemia is detected on stress testing, or if anginal symptoms interfere with the patient's quality of life or are new, coronary angiography may be recommended. There is significant variability regarding type of information delivered and how it is communicated by clinicians; decision aids are not currently utilized during the clinical encounter, although patients may be given an educational pamphlet about coronary angiogram and PCI, and often watch a video with technical details of the procedure. Common language observed included, “If there is a severe blockage, we can go ahead and fix it at that time.” We infrequently observed identification that there was a choice to be made or elicitation of patient values and preferences.

At our institution, patients who undergo coronary angiography typically do so on the day following consultation with a cardiologist, as many travel from a distance and request the convenience of next day scheduling. Informed consent is typically obtained by a cardiology fellow in the prep area of the catheterization lab and involves review of the risks, rather than potential benefits, of the procedure. There is not currently an opportunity to engage in shared decision making at this point for OMT vs. PCI+OMT, as the decision to proceed with coronary angiography with the possibility of PCI typically has already been made by the referring cardiologist with the patient; it is rare that patients expect to pause between diagnostic catheterization and intervention for shared decision making. Once the coronary anatomy is known, the interventional cardiologist will call the referring cardiologist on the phone while the sedated patient remains on the table. The interventional and referring cardiologist achieve consensus on whether to proceed with PCI, and the referring clinician follows the patient after procedure. For those patients in whom a choice of coronary artery bypass is relevant on the basis of diagnostic angiography, the decision aid is no longer applicable, as we have not modeled the choice of surgery at this time. Clinicians are encouraged at our institution to utilize a heart team approach and involve the cardiac surgeons, referring and interventional cardiologists, and the patient and their family, to select among PCI+OMT, PCI and coronary artery bypass surgery.

### Results Step 3: Development of Initial Prototype

**Figure 3:**
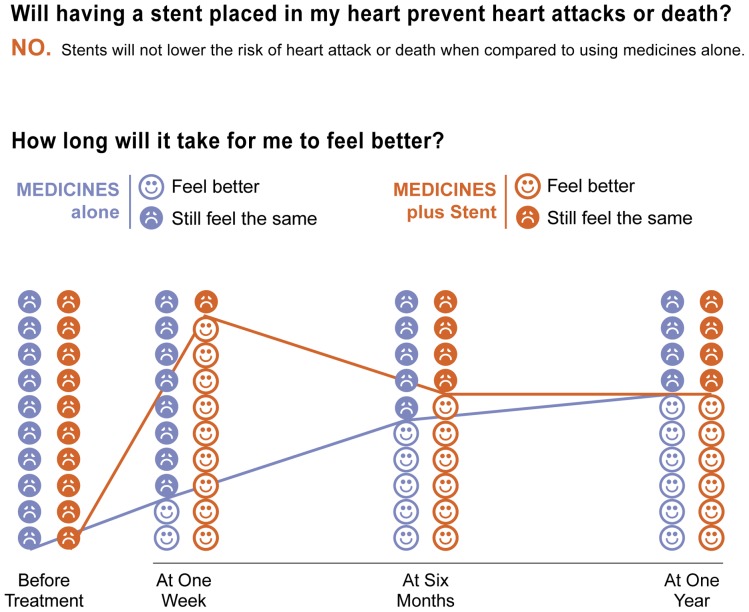
PCI Choice: early prototype of benefits page. Used with permission of Mayo Foundation for Medical Education and Research; Creative Commons License does not apply.

Two clinicians (H.H.T, and M.C.) partnered with a designer (K.S.) to create the first prototype. (Figure 3) Prominent in the initial design was modeling of benefit over time. Several delivery formats of the decision aid, PCI Choice, were considered, including desktop computer-based, handheld device-based, reusable plastic cards, and paper-based. We designed a one-page decision aid to confer portability, accessibility, scalability, and low cost features.

### Results Step 4: Field testing of initial prototypes

The initial prototype was first tested with the Diabetes Research Advisory Group.

Most strikingly, the group stated the tool did not appear to be a true “decision aid” based on the benefits page alone, as they felt the decision was a “no-brainer” to choose medical therapy. Similar quotes were found among clinic patients who were asymptomatic; this was in stark contrast to those limited by angina, reinforcing the central nature of symptoms to this specific patient-centered decision making process. Based on this, we created two distinct decision aids that offered more personalized estimates of benefit for patients depending on the severity of their baseline angina.

The Cardiovascular Patient and Family Advisory group was comprised of many individuals with a history of PCI or coronary bypass surgery, and here we identified the challenge of communicating the lack of mortality benefit for patients who had already undergone PCI, as many members of the group believed in a mortality benefit of stents for stable CAD. Further modifications based on concerns raised by this group were made to emphasize the relevance of PCI Choice for stable, as compared to unstable, CAD.

### Results Step 5: Successive iterations

In the real-word clinical encounters, over 20 patients with stable CAD were observed and interviewed while clinicians delivered iterative versions of the decision aid; 5 additional patients with a history of CAD were recruited from cardiac rehabilitation to provide feedback. We provided “just-in-time” training to the clinicians before the clinical encounter, reviewing the decision aid contents and recommending key concepts to reinforce.

Patients expressed an overwhelming preference for pictographs after we displayed benefit and risk in several ways, including shaded bars to depict relative differences between options, bar graphs in place of pictographs, and text-heavy descriptions. It became clear that the information central to the decision-making process was the benefit of PCI+OMT vs. OMT. This patient-based observation was striking, as risk was previously the focal point of discussions when considering PCI. Based on patient input, we added two sections: one on cross-over from medical therapy to PCI and another on risk of restenosis.

We found that excessive text limited the natural conversation between clinicians and patients, and thus focused on pictorial display. The number of graphs displaying risks was decreased due to a lack of patient interest in reviewing each individual risk. We inserted percentages next to the pictographs following observations of clinician difficulty with verbalizing graphical representation of data, which improved flow of the conversation. We placed a large-typed question at two points in the decision aid to lead both parties toward a discussion of patient goals and preferences.

**Figure 4:**
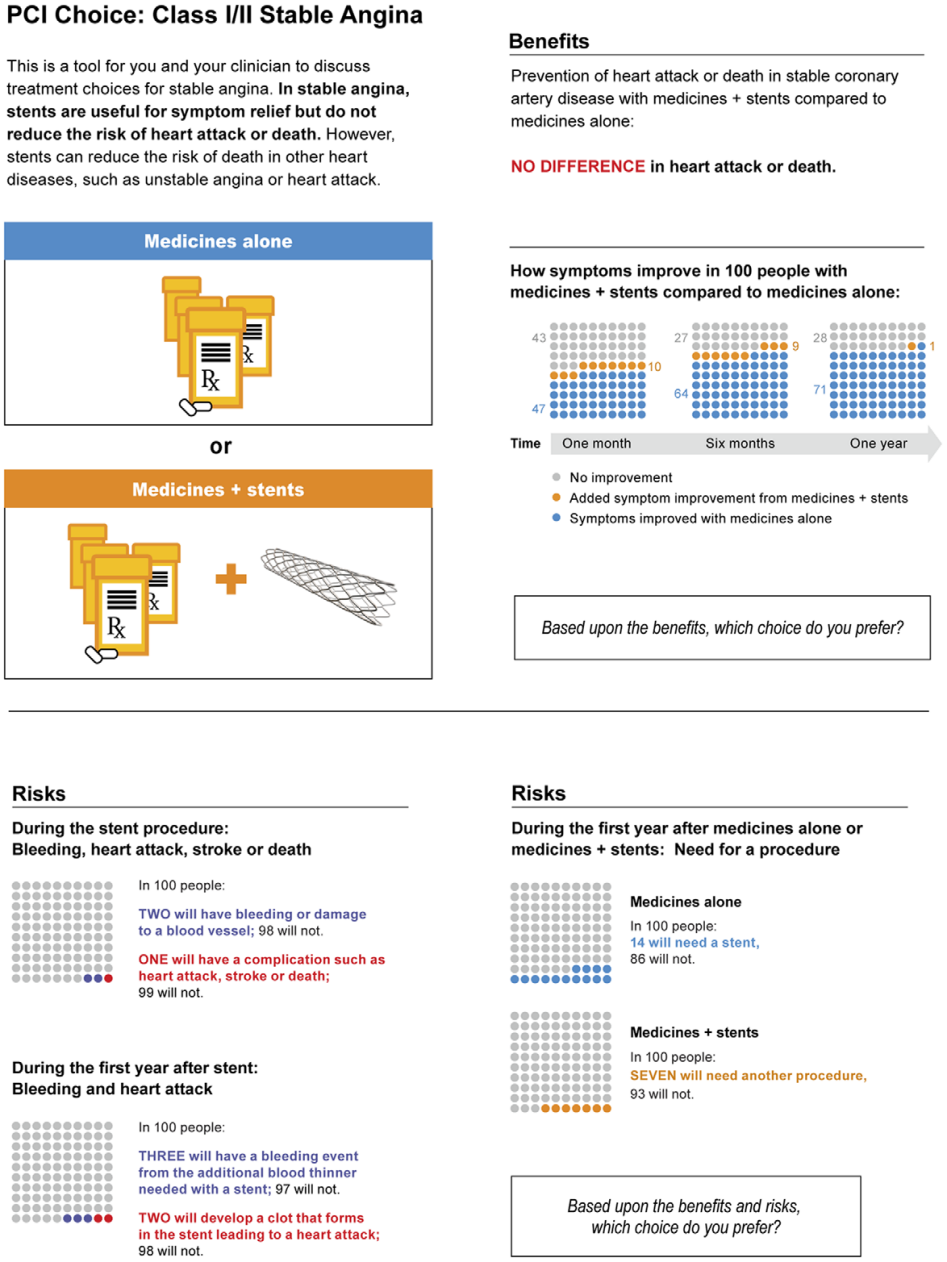
PCI Choice: final prototype. Used with permission of Mayo Foundation for Medical Education and Research; Creative Commons License does not apply.

The process of observing clinicians delivering the decision aid and directly interviewing patients regarding content and format of the decision aid was repeated over five months. Once the study team was satisfied, we met again with our patient advisory group, who approved the format and called the tool “enlightening.” Finally, we were confident that the tool was likely to consistently create effective conversations around treatment choices for stable CAD and lead to decisions that reflected both the research evidence and the values and preferences of the informed patient. (Figure 4)

## Discussion

Following the publication of comparative effectiveness research demonstrating no difference in death or MI with PCI+OMT for stable CAD compared to OMT, there has not been a substantial increase in the use of OMT [26]. Equally concerning is evidence of poor adherence with dual anti-platelet therapy following stent implantation, with the resulting risk in early to late stent thrombosis [27]. There is a growing literature base demonstrating overuse of coronary angiography and elective PCI [28], [29], along with a call for a “pause” of ad hoc PCI to improve shared decision making [30]. With PCI being a common procedure (622 000 in 2007) [31] performed at a considerable cost (greater than $12 billion annually) [32], its appropriate use is a national health care priority [33]. Previous work clearly outlines misconceptions by patients regarding benefits of PCI, identifying an existing gap in the standard informed consent process [4], [34]. We designed an individualized, patient-centered decision aid, PCI Choice, to assist clinicians and their patients considering PCI for stable CAD and to promote incorporation of patient values and preferences into decision making.

When examining the criteria for effective decision aids, PCI Choice addresses many of the key components of the International Patient Decision Aids Standards collaboration (IPDAS) including a systematic development process, presenting information on options and probabilities of outcomes, clarifying values, and using the scientific literature and patient stories on which to base the content, delivered in plain language [35].

Shared decision making tools can take many forms, including nurse-led group visits, personalized informed consent forms, videos, or decision coaches, among others [36], [37], [38]. For example, novel informed consent forms designed for use with patients undergoing angiography for stable angina successfully transfer knowledge about the mortality and bleeding risks associated with PCI and the benefit in target revascularization rates associated with drug-eluting vs. bare metal stents; these forms increase patient involvement in decision making across diverse sociodemographic groups [39]. However, decision aids, the type of tool used in this study, are distinct from traditional informed consent documents, with informed consent conventionally used once a treatment choice has been selected. Decision aids are designed as an adjunct to a conversation that is already occurring between patient and clinician, helping to clarify and reinforce key issues specific to the individual patient as clinicians and patients select a treatment choice. Finally, decision aids differ from patient education materials, which are heavily text-based, designed to be read outside the clinical encounter, and are not tailored to the individual circumstances of the patient.

### Conclusion

Significant misconceptions remain among patients with stable CAD regarding the benefits of PCI, a common, costly procedure that may be overused. In creating a patient-centered decision aid for stable CAD, we involved clinicians, health policy researchers, designers, patient focus groups and patients with stable angina to develop the best tool possible. We hypothesize that in an upcoming randomized trial, PCI Choice will lead to increased patient knowledge and patient involvement through effective translation of the best available comparative effectiveness evidence while incorporating patient values and preferences.
